# Wireless Power-Up and Readout of Label-Free Nanosensors for In-Vivo Monitoring of Protein Concentrations in Live Animals

**DOI:** 10.1021/acs.langmuir.5c01350

**Published:** 2025-09-12

**Authors:** Hassan Raji, Suneel Kumar, Zhuolun Meng, Francois Berthiaume, Mehdi Javanmard

**Affiliations:** † Department of Electrical and Computer Engineering, 5970 Rutgers University, Piscataway, New Jersey 08854, United States; ‡ Department of Biomedical Engineering, Rutgers University, Piscataway, New Jersey 08854, United States

## Abstract

Tracking inflammatory biomarkers in real-time is essential for timely clinical interventions; however, wired sensors are restrictive, inconvenient, prone to infection risk, and limit continuous monitoring compared with wireless sensors. In this study, for the first time ever, to the best of our knowledge, we report a novel method for wireless power-up and readout of a label-free electronic biosensor for quantification of protein biomarkers in live animals. The sensor operates through resonant inductive coupling for wireless power transfer, enabling remote impedance measurements of a nanowell array without requiring a direct electrical connection. The receiver circuit is integrated within a 3D-printed structure optimized for application on wound sites. Experimental validation included titration of IL-6 across a wide concentration range and in vivo testing on 30 animals with induced wounds. The wireless sensor’s measurements showed a strong correlation (*R* ^2^ > 0.9) with standard ELISA results. This platform offers a noninvasive, real-time, portable approach to tracking inflammation, potentially improving wound care management and patient outcomes.

## Graphical abstract

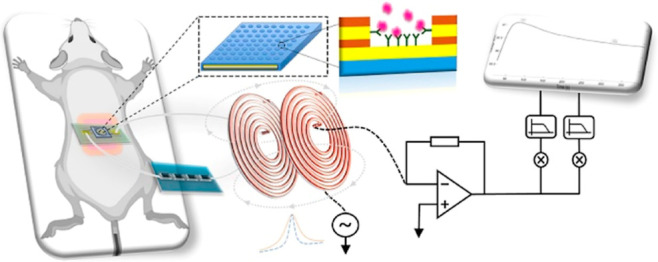

## Introduction

Chronic wounds pose a significant healthcare challenge due to delayed healing, infection risks, and systemic complications.^1,2^ Improperly healing wounds can cause pain, reduced mobility, chronic inflammation, infection risks, systemic complications like sepsis, emotional distress, and financial strain on patients and healthcare systems.^3,4^ Monitoring wound healing is crucial for assessing tissue repair, detecting complications, and evaluating treatments.^5^ Biomarkers play a key role in disease diagnosis, progression monitoring, and treatment evaluation.^6–10^ IL-6, as an inflammatory marker, provides insights into wound healing by indicating inflammation status and aiding in early intervention.^11^ Traditional methods for measuring inflammatory markers are limited by long processing times and specialized requirements, making them unsuitable for real-time or point-of-care applications.^12,13^ In contrast, real-time and continuous monitoring improves early complication detection, treatment adjustments, and patient outcomes while reducing healthcare costs.^14^ This highlights the need for highly sensitive, real-time, and point-of-care detection methods with minimal sample preparation.^15^

Miniature biosensors enable rapid and precise detection with minimal sample volumes, enhancing efficiency in analytical applications.^16–18^ Label-free detection minimizes sample preparation, preserves biological integrity, and allows real-time, high-sensitivity monitoring,^19–21^ whereas label-based detection introduces complexity through additional steps and potential biomolecular interference.^22,23^ Optical methods provide molecular details, but label-free electronic biosensors are more suitable for point-of-care applications due to their ease of miniaturization, minimal reagent use, and real-time capabilities.^24,25^ Nonfaradaic impedance biosensors, while practical for point-of-care diagnostics, face challenges in sensitivity and data interpretation.^26–28^ High salt concentrations and complex biological samples can further affect sensor performance.^29^ Therefore, structural design should ensure uniform antibody immobilization, geometric confinement of binding interactions, localized detection, and an optimal surface-to-volume ratio for enhanced sensitivity.^30,31^ Additionally, the sensor should enable real-time monitoring, rapid and accurate responses, and reliable signal detection in noisy environments.^32^ The sensitivity of impedance-based sensors is limited by electrode spacing, necessitating a reduced distance to strengthen the electrical field and improve detection performance.^33^

Wireless power transfer (WPT) enhances biosensor applications by providing mobility and portability, enabling untethered operation in modern biomedical technologies.^34–36^ Unlike wired systems, WPT allows seamless functionality in wearable and implantable devices, improving flexibility and usability across diverse biomedical settings.^37–39^ Wireless biosensors facilitate continuous biomarker monitoring, offering advantages such as mobility, reduced contamination risk, and improved patient comfort, particularly in tracking IL-6 for early sepsis detection and real-time therapeutic guidance.^40–43^ By eliminating physical power connections, WPT reduces biosensor cost and complexity while enhancing reliability, durability, and long-term usability compared to battery-powered alternatives.^44–46^ It also avoids complications associated with surgery-based treatments, making it a safer and more practical solution.^47^ These advantages support diverse applications, including disease diagnosis, health monitoring, and vital sign tracking.^48^ Inductive coupling WPT enables noninvasive data collection and efficient operation at low input voltages, though its performance is highly dependent on precise coil alignment.^49–51^ To ensure uninterrupted sensing during patient mobility, systems must be designed to minimize misalignment and maintain consistent power transfer.

To address the limitations of the previous miniaturized sensor,^17^ we developed a novel biosensor design in this work that ensures precise alignment of the two coils, enabling effective in vivo testing on live animals. The earlier design faced challenges due to size disparities between components, compromising sensitivity, reliability, and repeatability. In this work, we employed a resonant inductive coupling system using RFID coils as both transmitter and receiver, supported by a custom 3D-printed structure designed to house the receiver circuit and facilitate its use in animal studies. The sensing region of the nanowell sensor array, created between two overlapping electrodes using nanofabrication techniques, is positioned within the receiver circuit. The experiments differentiated the target protein from the negative control and characterized the sensor’s response to varying protein concentrations, allowing for the extraction of the titration curve. A rigorous in vivo study involving 30 animals was then conducted to measure IL-6 levels in wound sites, with ELISA assays performed on collected wound samples to validate and compare the results obtained with the wireless biosensor, confirming its sensitivity and reliability for biological applications.

## Results

### Extraction of the Standard Titration Curve

The full experiment procedure to detect target biomarker is detailed in Section 1.3 of the Supporting Information. A schematic of the experiment procedure is shown in Figure 1.

**Figure 1:**
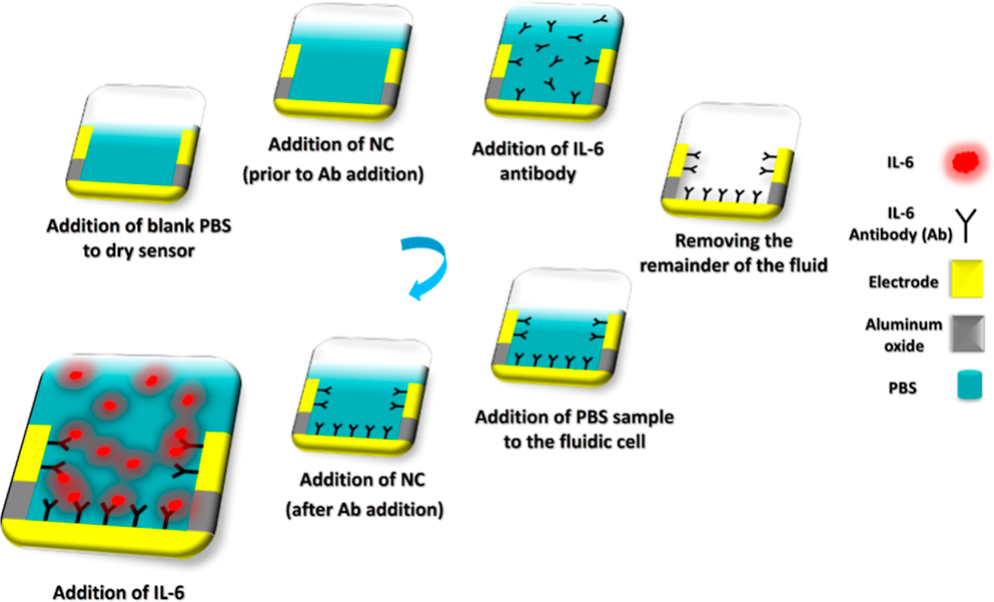
Experiment procedure to detect IL-6.

The quantification of biomarkers provides critical indicators for monitoring disease progression, evaluating treatment efficacy, and gaining insights into pathology models.^52^ IL-6 plays key roles in the acute phase response, inflammation, hematopoiesis, bone metabolism, and cancer progression, and serves as a biomarker for diseases including rheumatoid arthritis, sepsis, and cancer.^53^ In this respect, a titration curve is obtained using standard concentration samples of mouse IL-6 with the wireless sensor to achieve this. The titration curve provides valuable insights into how the sensor responds sensitively to varying concentrations of this important biomarker. The titration curve help derive a regression model that can later quantify IL-6 in mouse wound fluid samples, with the results being compared to ELISA measurements of the collected wound fluid samples. Experiments are carried out to determine how IL-6 concentration correlates with sensor response. The study tested five concentrations: 500 pM, 5 nM, 50 nM, 500 nM, and 5 μM. Each concentration was tested in triplicate to establish error bars. The purpose of these experiments is to validate sensor performance by monitoring impedance. All measurement steps, except for antibody immobilization, are completed within approximately 10 min each. For the antibody step, the incubation time typically ranges from 10 to 20 min until the output signal reaches a plateau, as shown in Figure S3c. The experimental protocol for all trials began with adding 10 μL of blank PBS to the dry sensor. Next, 2.5 μL of IL-6 antibody was applied, resulting in a gradual signal decrease. A 2.5 μL negative control blank PBS sample was then introduced, causing the signal to progressively increase, in contrast to the antibody’s effect. After removing the remaining fluid from the sensor, 10 μL of PBS was added, followed by another 2.5 μL of negative control blank PBS, which again led to a gradual signal increase. Finally, 2.5 μL of IL-6 was added to the sensor. Using the wireless sensor, the response at each concentration was quantified. Figure 2 illustrates the Titration-based analysis of IL-6 detection using the wireless biosensor. Using MATLAB curve fitting, the titration data in Figure 2C was fitted with a rational (2/2) regression model, yielding an *R* ^2^ of 0.986 and an RMSE of 0.121, confirming the strong concentration-dependent response of the sensor.

**Figure 2:**
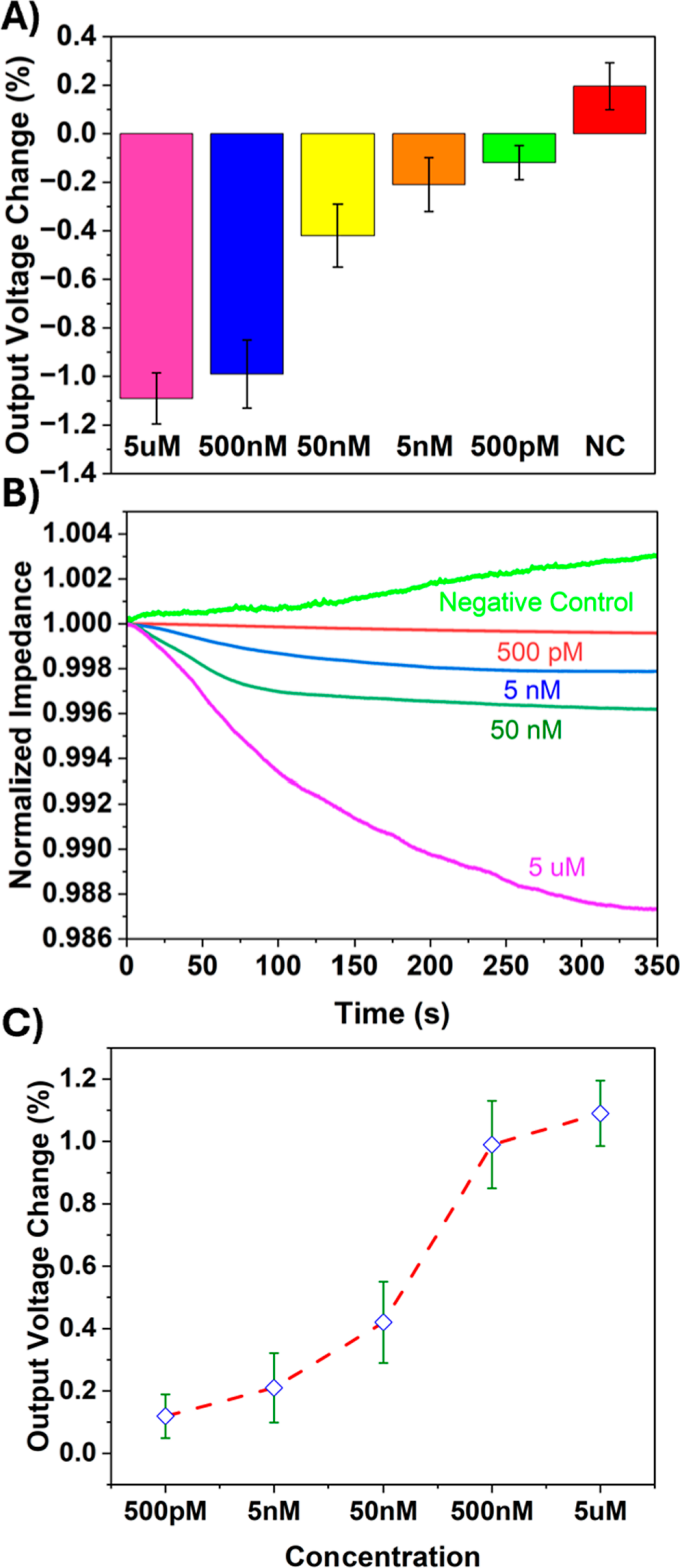
Titration-based analysis of IL-6 detection using the wireless biosensor: (A) Comparison of the average gradual change in triplicate titration experiments for different IL-6 concentrations and the negative control. Error bars represent the standard deviation. (B) Output voltage variation over time for different IL-6 concentrations and the negative control, demonstrating a concentration-dependent response (See Figure S6 in the Supporting Information, highlighting lower concentration differences). (C) Titration curve illustrating the wireless biosensor’s sensitivity to varying IL-6 concentrations.

All measurements were performed inside a Faraday cage to effectively isolate the system from external electromagnetic interference and minimize environmental noise. Minor sensor drift was observed, mainly due to evaporation inside the nanowells, but this was clearly distinguishable from the gradual, time-dependent response associated with antigen–antibody binding. To minimize electrode fouling and maintain consistent sensor performance, we applied standardized washing steps after antibody incubation as discussed, and the nanowell geometry limited exposure to only the sensing area. A protective oxide layer further minimized surface contamination. In addition, we applied consistent sample preparation protocols and, where applicable, employed normalization strategies to enhance comparability.

The titration curves for IL-6 demonstrate the biosensor’s ability to detect protein concentrations with accuracy across a broad range, showcasing its potential in biomedical applications. Precise biomarker detection is critical for disease diagnosis and monitoring, and this sensor technology addresses that need effectively. The experiments revealed a gradual baseline shift in impedance plots as IL-6 concentration increased. This shift is attributed to the binding interactions between IL-6 and its specific antibodies. At a low concentration of 500 pM, the impedance shift closely resembled the response of the negative control.

### Comprehensive Animal Experiment

Examinations of live animal models allow data collection under realistic physiological conditions, enhance biological relevance, support preclinical studies, and validate sensors for monitoring wound progression and detecting biomarkers in wound fluid. In our previous study, extensive in vitro and in vivo evaluations were conducted to confirm the biocompatibility of the sensors before initiating animal experiments. Therefore, we designed a comprehensive animal experiment with 30 animals to measure wound IL-6 within wound fluid. Figure 3A–C illustrates an animal at three stages: before wound creation, after wound creation, and after placing the sensor on the wound during measurement.

**Figure 3:**
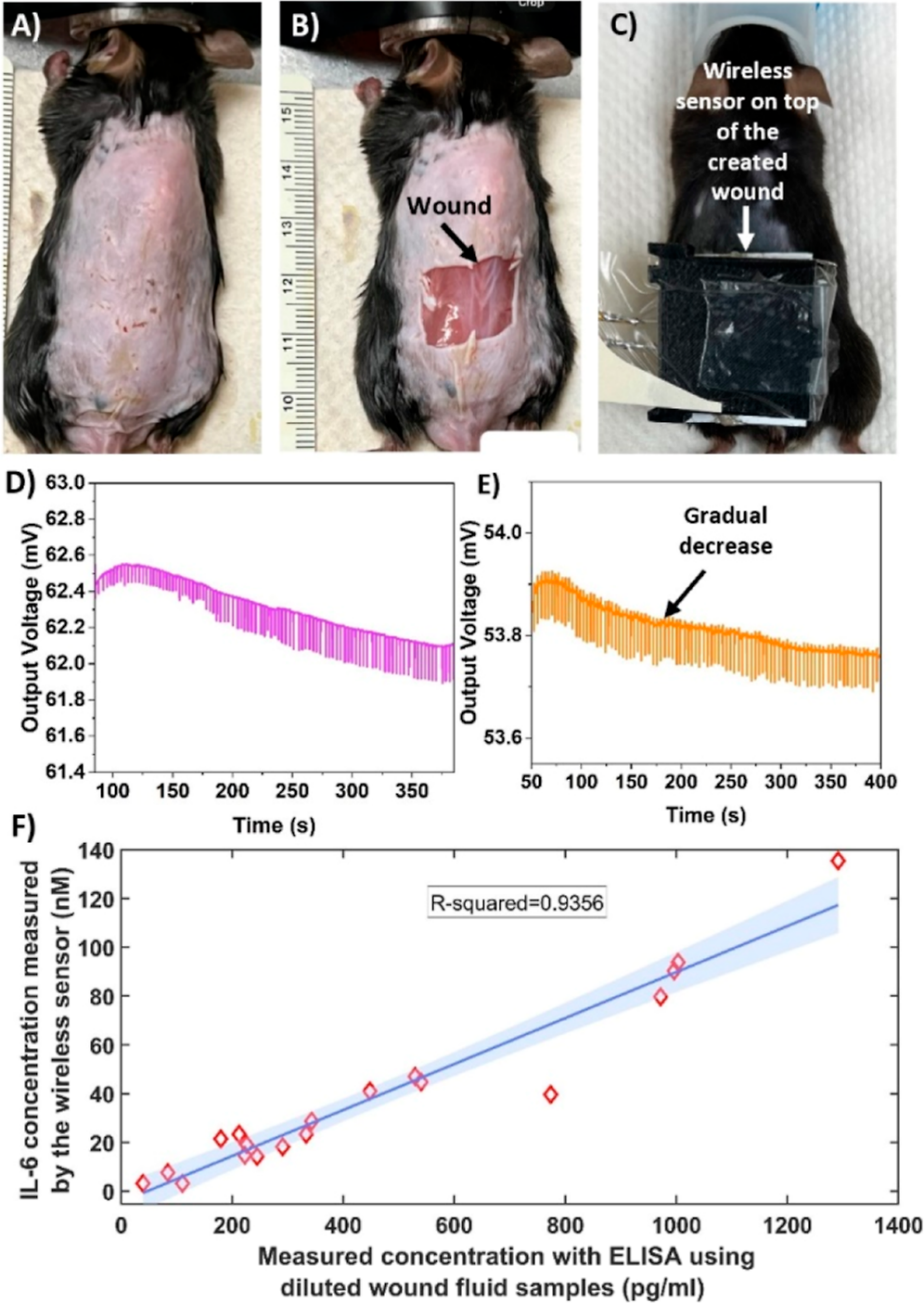
Validation of animal wound measurements. (A) Animal prior to wound creation; (B) animal following wound creation; (C) animal during measurement with the sensor positioned on the wound; (D,E) wireless sensor output voltage during IL-6 detection in wound fluid for two different animals; (F) comparison of IL-6 concentrations obtained from sensor measurements in the wound and ELISA analysis of collected wound fluid samples. The 95% confidence interval for the fitted line has been included and is visually represented as the shaded blue region around the regression line.

All animal procedures were conducted with the approval of the Rutgers Institutional Animal Care and Use Committee (IACUC). We purchased C57BL/6 male wild-type mice (9 weeks old, weight = 25 g) from Charles River Laboratories (Wilmington, MA) and allowed them 1 week for laboratory conditions acclimatization. 24 h before the experiment, mice were anesthetized with isoflurane (Henry Schein, Melville, NY), shaved, and treated with a depilatory agent (Nair Cream) to ensure complete dorsum hair removal. On the surgery day, mice were anesthetized similarly, and their dorsum was sterilized using betadine scrub and 70% ethanol thrice alternatively. A full-thickness wound measuring 1.5 cm × 1.5 cm was then created on the back of each mouse. The wound was covered with Tegaderm transparent dressing and removed it to collect the consistent wound fluid. The Tegaderm dressing was then rinsed with 1 mL of PBS immediately after its collection, and the collected wound fluid solution was frozen immediately for later analysis. Images of the eight other animals are provided in Figure S5. The sensor is prepared following the same protocol used in our previous biocompatibility experiments, ensuring sharp edges are smoothened, and the sensor is sterilized before advancing to further studies. The experimental procedure closely follows the approach outlined in prior sections with a key modification, where the functionalized sensor is placed directly over the wound instead of introducing the target antigen to the sensor. After the sensor is positioned on the wound, the opposite side of the 3D-printed housing is secured with tape to keep the sensor stable during measurements. Real-time impedance measurements are performed concurrently to monitor and analyze the binding of the target antigen over time. Figure 3D,E presents the time-series output voltage data from the lock-in amplifier for two of the animals. The impedance exhibits a similar decreasing trend over time, consistent with the findings in previous sections and the measurements obtained using our wired sensor. This pattern is indicative of the time-dependent antigen–antibody binding behavior. This gradual change is represented as a percentage change to quantify the concentration of antigen in the wound fluid. The time-series data also reveals small pulses attributed to the animal’s breathing during the measurements. Upon completing the experimental procedure for 19 animals, the PBS rinse collected from the wound is analyzed offline using commercial enzyme-linked immunosorbent assay (ELISA) kits (Invitrogen, Thermo Fisher Scientific). Figure 3F shows the quantified measurements of the wireless sensor using the titration curve and ELISA measurements with an R-squared of 0.9356. The normalized RMSE was 0.0991, further indicating strong agreement between the two methods. The regression analysis yielded a slope of 0.0943 (95% CI: 0.0816–0.1069), indicating a consistent linear relationship between ELISA and sensor measurements, while the intercept of −4.43 (95% CI: −11.87 to 2.99) was not significantly different from zero. This comparison highlights the high accuracy and reliability of our sensor in quantifying IL-6 levels, as the results strongly correlate with those obtained through ELISA, which is widely regarded as the gold standard for biomarker quantification. A key benefit of our sensor is its portability, which enables on-site and point-of-care testing, whereas ELISA systems lack this capability and require dedicated laboratory facilities. Unlike ELISA, which requires several hours to complete, our sensor delivers results in real-time, enabling rapid decision-making in critical applications. Furthermore, the sensor requires minimal sample preparation, significantly reducing the complexity and time associated with the testing process. In contrast, ELISA necessitates multiple sample preparation steps, which can introduce variability and extend the testing duration. Additionally, our method requires a substantially smaller volume of reagents, offering a cost-effective and resource-efficient alternative to ELISA.

## Conclusions

This work presents a significant advancement in wireless, in vivo, label-free, continuous biomarker sensing technology on live animals, with a particular focus on monitoring inflammatory markers such as IL-6 in wound healing applications. The sensing component is fabricated through a series of nanofabrication processes applied to multiple layers, resulting in two opposing electrodes separated by a narrow 40 nm gap. The overlapping region of these electrodes undergoes multiple etching steps to form a 5 × 5 array of wells, each with a diameter of 2 μm, thereby establishing a nanometer-scale pathway between the electrodes. This innovative approach is particularly significant, as alternative well-based sensors typically feature micrometer-scale distances between electrodes, which weaken the electric field and, consequently, reduce measurement sensitivity. Additionally, the array format of this sensor enhances sensitivity by enabling the simultaneous detection of multiple interactions and amplifying the overall signal response. Introducing antibodies or antigens results in their physical immobilization on the electrode surface, obstructing the ionic current and leading to a measurable change in impedance.

By integrating a nanowell impedance array with a resonant inductive coupling system for wireless power transfer and readout, we have addressed critical challenges in traditional, wired biosensing methods and established a new paradigm for point-of-care diagnostics. The receiver side incorporates modulation components to align the resonance frequencies of both sides, thereby maximizing energy transfer. This enables the nanowell array’s binding events to be detected through a lock-in amplifier that measures the net impedance on the transmitter side. Additionally, we designed a 3D-printed structure to efficiently house the entire wireless sensor, allowing it to function as a wearable device with minimal modifications. This further enhances the portability of the sensor while ensuring the stability of the setup, even during in vivo measurements and continuous biomarker monitoring as a wearable device. Our sensor design, which includes RFID-based receiver and transmitter coils, precise nanowell geometry, and durable 3D-printed housing, delivers stable, accurate, and highly sensitive protein biomarker detection under real-world conditions, including in vivo testing on animal wound models. The successful demonstration of wireless, real-time IL-6 monitoring validates both the sensitivity and reliability of our wireless label-free sensor. A clear distinction was observed in the measured signal between the target antibody/protein and the negative control, as well as between different concentrations of IL-6. This enabled further experimentation with multiple IL-6 concentrations to derive a standard titration curve. The platform’s ability to differentiate target proteins from negative controls, generate reproducible titration curves over a wide dynamic concentration range, and track subtle changes in impedance caused by biomolecular interactions underscores its robustness and versatility.

We performed a comprehensive animal experiment to quantify IL-6 within the wound fluid of live animals. Our experiments confirmed that the sensor’s response strongly correlates with standard ELISA measurements (*R* ^2^ > 0.9), affirming its quantitative accuracy. By achieving high fidelity in complex biological environments, the system offers tangible clinical benefits. It provides continuous monitoring of wound inflammation levels without the need for invasive procedures or labor-intensive assays, paving the way for more proactive and personalized patient care. Furthermore, our sensor offers accuracy comparable to gold-standard methods such as ELISA while being portable, delivering rapid sample-to-answer results, requiring minimal reagents, and featuring straightforward experimental procedures, advantages not typically attributed to ELISA.

From a clinical translation standpoint, the portability and wireless operation of our system eliminate many of the logistical obstacles associated with conventional biomarker detection methods. The wireless design allows for a compact, wearable form factor that can be adapted for a range of clinical scenarios, and the capacity to integrate into existing wound dressings or medical devices further enhances patient compliance and comfort. Moreover, by obviating the need for bulky optical components, labeling reagents, or complex sample preparation, our approach reduces time, cost, and labor requirements, ultimately making advanced monitoring more accessible in resource-limited settings or in decentralized healthcare environments. The wireless system offers a form factor advantage over battery-powered alternatives by eliminating bulky batteries and reducing associated thermal and safety concerns. Although a benchtop lock-in amplifier was used in this study, its function can be integrated into a compact circuit, supporting future development of a fully portable system.

Our findings have broad implications for wound care management and beyond. Continuous, label-free tracking of IL-6 and other inflammatory cytokines holds the potential to transform both acute and chronic wound assessment, enabling early detection of complications such as infection or chronic inflammation. Clinicians could leverage this information to intervene promptly, tailoring treatment protocols in near real-time and improving healing outcomes. Beyond wound healing, the same technology could be applied to monitor a variety of biomarkers associated with other pathological states—ranging from metabolic disorders to cardiovascular conditions—where real-time, on-site data acquisition can significantly improve patient management.

In essence, this work lays down a solid foundation for the future of wireless biosensors that merge advanced nanofabrication, resonant inductive coupling, and streamlined packaging. By achieving high sensitivity, specificity, reliability, and ease of integration into practical healthcare scenarios, we have taken a crucial step toward realizing truly continuous and noninvasive patient monitoring. This approach opens new avenues for precision medicine and telemedicine, where ongoing assessment of protein biomarkers can be seamlessly integrated into clinical workflows, improving diagnostics, guiding therapy, and ultimately enhancing patient care.

This study highlights the transformative potential of wireless biosensor technologies in advancing wound care management and real-time health monitoring. By providing noninvasive, portable, and efficient diagnostic tools, the proposed system paves the way for improved clinical decision-making and better patient outcomes. We note that additional negative controls, such as BSA or nontarget interleukins, are required to be examined in future studies, which were not included in the present work. Future work could focus on optimizing the system for detecting a broader range of biomarkers and exploring its applicability in other medical conditions to further expand its utility in precision medicine.

## Materials and Methods

### Overview of the Wireless Setup and 3D-Printed Housing

The nanowell array features two electrodes separated by a 40 nm dielectric oxide layer, enabling impedance measurement and overcoming the challenge of weak electric fields observed in previously reported impedance biosensors. Schematic of and fabrication of the nanowell array is shown in the Figure 4.

**Figure 4:**
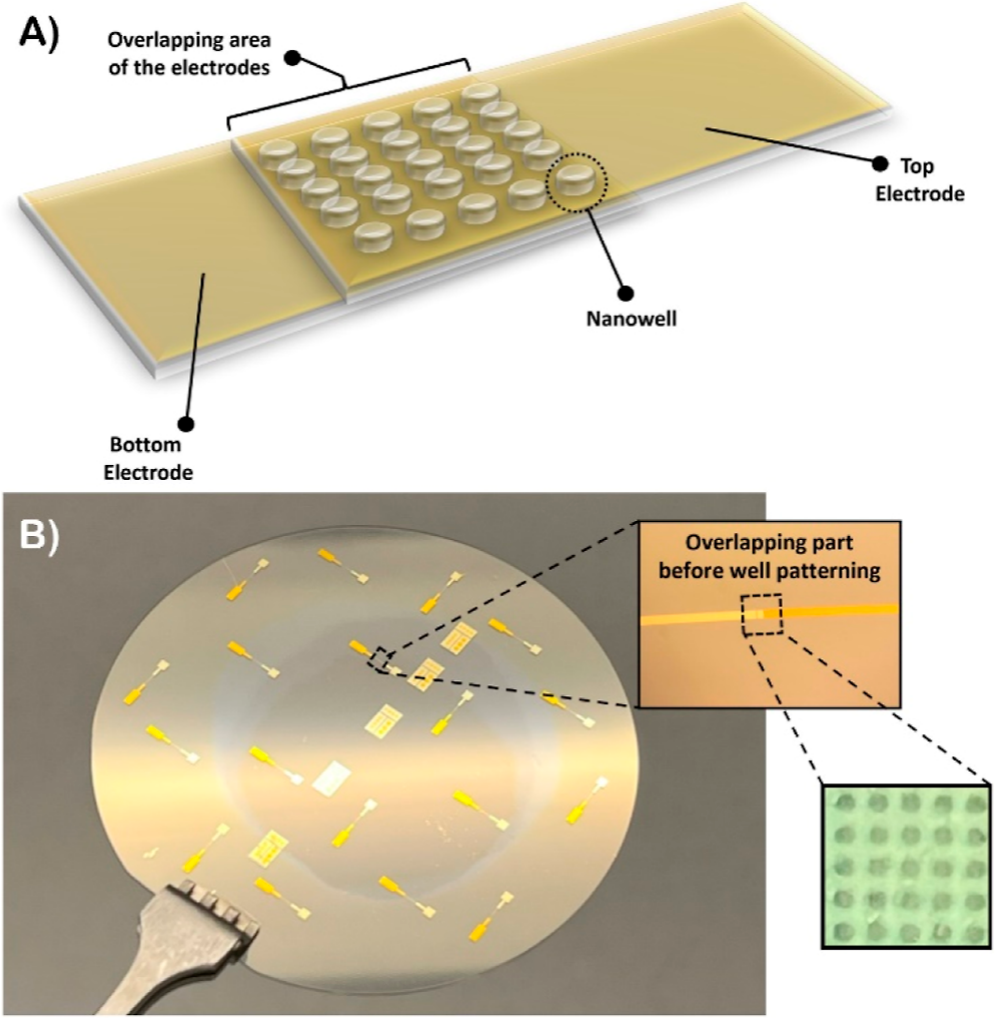
Schematic of nanowell array and fabrication: (A) schematic showing the nanowell array in the overlapping region of the top and bottom electrodes; (B) wafer containing multiple patterns of the two electrodes, each with an overlapping region where the nanowell array is patterned using multiple etching steps.

The fabrication of nanowell array is discussed in the Supporting Information (Section S1.1). The sensing component is designed in an array format, significantly enhancing sensitivity by increasing the surface-to-volume ratio, providing a larger interface for biomarker interaction, and enabling high-throughput detection, making it crucial for accurately identifying small quantities of biomarkers. Each nanowell in the biosensor features a conductive pathway formed between two overlapping electrodes, enabling precise probing of antibody immobilization within the well. The sensing system comprises two inductively coupled coils: a transmitter and a receiver. The receiver coil, consisting of 13 turns with a diameter of 2.17 cm, is connected in series with modulating components and the nanowell array, forming the receiver circuit. The transmitter and receiver are not physically connected; instead, modulating components are used to align the receiver’s resonance frequency with that of the transmitter. This alignment ensures effective resonant inductive coupling, as both circuits are configured to achieve a matching resonance frequency with the measurement frequency. This enhances energy transfer efficiency, facilitating optimal power delivery and improved sensitivity for precise and reliable sensor performance. Impedance spectrum and resonance frequency measurements are carried out using the Impedance Analyzer (Keysight Technologies E4990A IA, CA, USA) and the Impedance Spectroscope (Zurich Instruments HF2IS, Zurich, SI). During measurements, the transmitter coil is positioned above the receiver coil, with precise alignment ensured by the design of the 3D-printed housing, as discussed in a later section. The transmitting component features an identical coil and links to a lock-in amplifier, gauging the real-time complex net impedance to identify biological binding events with 400 mV input, aligning with the measured resonance frequency corresponding to the receiver’s resonance frequency (27.6 MHz), with the power transfer efficiency of ∼48%. The transmitter coil is connected to the input at one terminal, while the other terminal links to a lock-in amplifier, which calculates the real and imaginary impedance components in real time. The system’s AC excitation source is configured to deliver 400 mV at a frequency near the resonance point. The excitation voltage serves as a reference in the circuit, with the response processed through several amplification and filtering stages. We specifically optimized the lock-in amplifier parameters for this application to maximize signal-to-noise ratio and resolution. The lock-in amplifier is configured with a 1 k gain, a 225 samples-per-second sampling rate, and a 2 Hz bandwidth. Impedance changes within the nanowells are detected using the impedance spectroscope via the lock-in amplifier. Variations in the nanowell array’s impedance are monitored in real-time by observing corresponding shifts in the equivalent impedance measured at the transmitter side. The overview of the wireless setup is shown in Figure S2 of the Supporting Information.

Ensuring a stable structure for animal experiments necessitated a design capable of housing all components of the receiver circuit without being fragile. Additionally, we engineered the packaging to be easily transformable into a portable wearable device with minor adjustments. Our design features a 3D-printed structure dedicated to accommodating the receiver circuit (i.e., wireless sensor). The structure is shown in Figure 5A–C.

**Figure 5:**
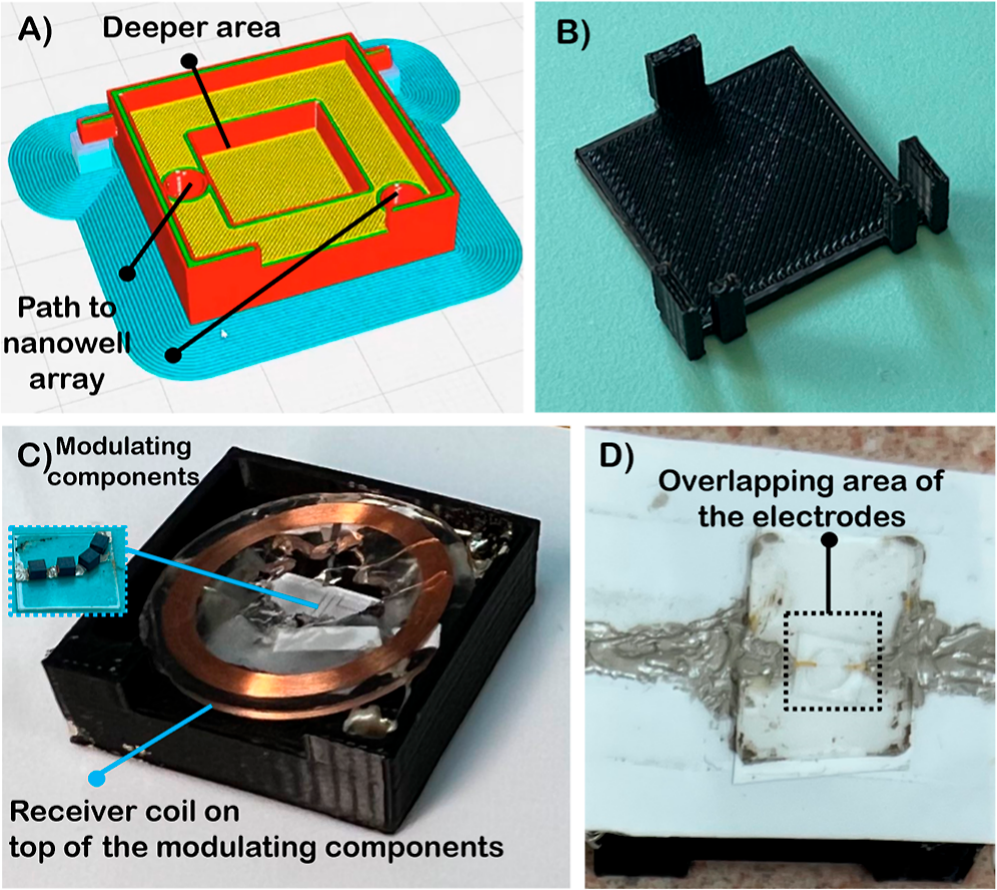
Overview of the 3D-printed packaging of the wireless biosensor: (A) first part of the structure, housing different components of the receiver circuit; (B) second part of the structure, enclosing the setup used during measurements; (C) top view of the first part of the structure, where the modulating components are initially placed in the deeper area before positioning the receiver coil on top; (D) back view of the structure, showing the nanowell array placed on a double-sided adhesive and epoxied to the other components of the receiver circuit.

This structure is designed in SolidWorks with minor modifications in the interface Ultimaker’s software. The housing was manufactured through 3D printing using an Ultimaker S5 printer with Ultimaker’s polylactic acid (PLA) filament (Utrecht, Netherlands). This structure comprises two sections. The first section houses all components of the receiver circuit (See Figure 5A,C). Figure 5A provides an overview of the first part of the structure, showing where the compartments are connected and where the modulating components are placed in the deeper area. On one side of this section, the modulating components are positioned atop square-shaped glass pieces precut using a laser cutter (See Figure 5A,C). This design allows for the secure placement of the glass within the structure. A larger square area, nearly matching the outer diameter of the RFID, is designated for positioning the receiver coil. The nanowell array is situated on the opposite side of this part of the 3D structure (See Figure 5D). We integrated two holes to facilitate the connection between the two sides of the nanowell array, the receiver coil, and the modulating components. All compartments of the receiver circuit are interconnected using conductive epoxy (Chemtronics CW2400). The transmitter coil is installed within the structure solely to enable measurements aligned with the receiver coil. The second part of the 3D structure can be positioned at this juncture to commence the measurement process (See Figure 5B). Figure 5C shows the structure after printing, with the components of the receiver circuit positioned and placed inside the structure. We utilize double-sided adhesive tapes (3 M 9965/9969 Diagnostic Microfluidics tape, 3 M, MN, USA) underneath the nanowell array, so it can be placed firmly on one side of the structure. Then, the interconnecting pads are epoxied to receiver coil and modulating components.

## Supplementary Material



## Notes

Additional details on fabrication of the nanowell array, details of the experimental procedure, extended discussion of results are provided in the Supporting Information (PDF)
