# Supplementary material for: Wireless Power-Up and Readout of Label-Free Nanosensors for In-Vivo Monitoring of Protein Concentrations in Live Animals
Source: Langmuir. 2025 Sep 12;41(37):25100–7. doi: 10.1021/acs.langmuir.5c01350 (PMC12461922; doi:10.1021/acs.langmuir.5c01350)
Supplement: Supplementary file 1 [file la5c01350_si_001.pdf]

Wireless Power-up and Readout of Label-free Nanosensors for In-vivo Monitoring of Protein Concentrations in Live Animals

Hassan Raji[a], Suneel Kumar[b], Zhuolun Meng[a], Francois Berthiaume[b] and Mehdi Javanmard*[a]

[a] Department of Electrical and Computer Engineering, Rutgers University, Piscataway, NJ, 08854, USA.

[b] Department of Biomedical Engineering, Rutgers University, Piscataway, NJ, 08854, USA.

1.1. Fabrication of the Nanowell Array

A fused silica wafer (University Wafer, South Boston, MA, USA) is cleaned using oxygen plasma, followed by photolithography patterning of the first electrode layer using AZ5214 photoresist. Fig. S1 shows the fabrication process of the nanowell array.

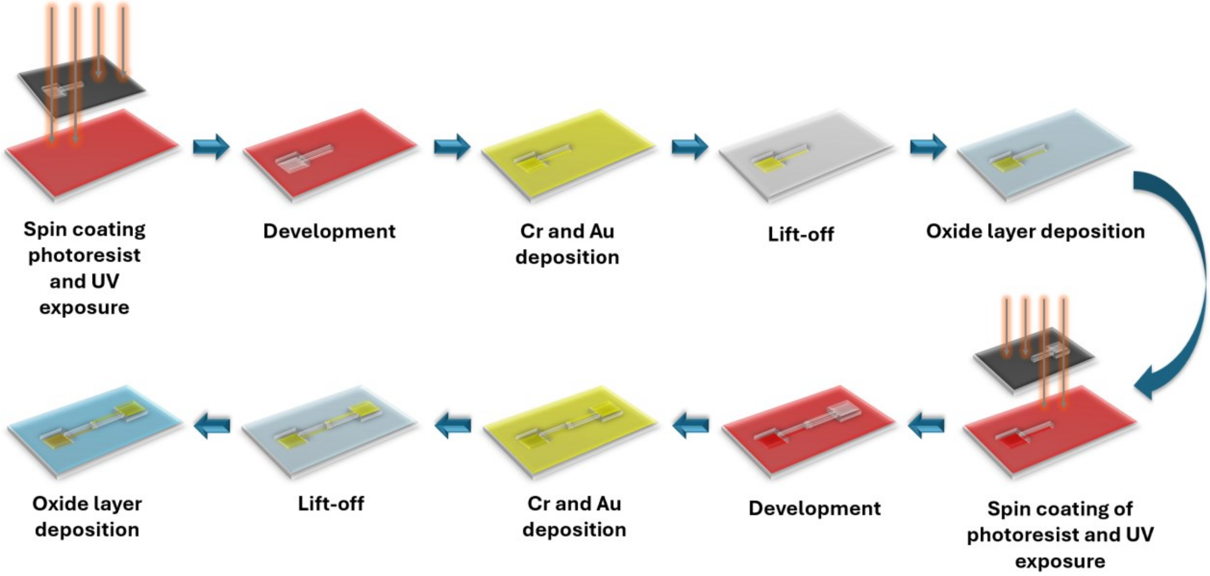

Fig. S1: Fabrication process of the nanowell array in which schematic illustrating all the steps required for fabricating the nanowell array.

In the next step, a 5 nm chromium layer is deposited using Ebeam Evaporation on the substrate as an adhesion layer before depositing 100 nm of gold. Lift-off is then used to pattern one of the overlapping electrodes, which is positioned beneath the other electrode, as explained subsequently. On top of the patterned electrode, a layer

of 40 nm is deposited using Atomic Layer Deposition (ALD) as an insulator layer between the two electrodes. The top electrode, connection pads, and interconnecting lines linking the electrodes to the pads are fabricated using a similar approach. An additional 40 nm aluminum oxide layer is precisely patterned over the top electrode in a subsequent fabrication step. The next step involves patterning the nanowell array in the overlapping area of the two electrodes. AZ5214 photoresist is spin-coated and patterned using photolithography. Wet etching is carried out to sequentially remove the oxide layer, gold layer, chromium layer, and an additional oxide layer within the well. This process utilizes buffered oxide etchant for the oxide layers, gold etchant for the gold layer, and chromium etchant for the chromium layer. Fig. 4(A) of the manuscript presents a schematic of the resulting nanowell array located in the overlapping area of the electrodes, and Fig. 4(B) of the manuscript displays a wafer containing multiple patterns. After stripping off the photoresist, a mask is used to pattern a new photoresist layer, enabling the selective removal of alumina from the non-sensing regions, resulting in a sensor with two overlapping electrodes. The wafer, containing multiple patterns of nanowell arrays, is divided into smaller sections using a laser cutter (Boss LS1420, MinPower (%)-1: 30, Max-Power (%)-1: 30, Speed(mm/s): 8, Boss Laser, FL, USA), ensuring that each section includes a single nanowell array along with the electrode pads. A 5mm circular PDMS well (SYLGRADTM 184 Silicone Elastomer) is bonded using oxygen plasma cleaner (Harrick Plasma PDC001HP, Ithaca, NY, USA) directly on top of the nanowell array.

1.2. Overview of the wireless setup and 3D-printed housing

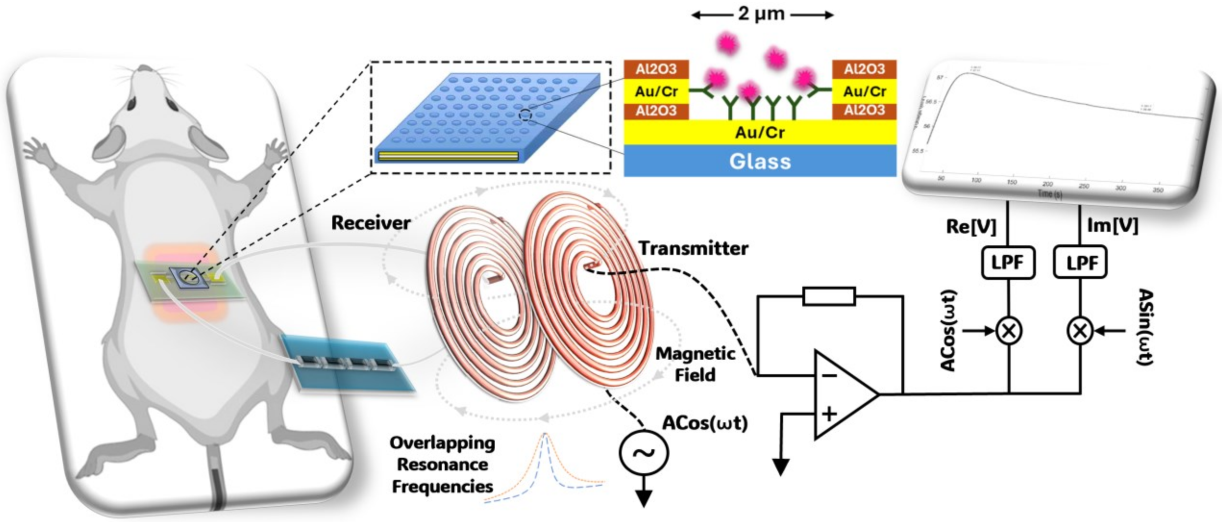

Fig. S2: Overview of the wireless setup for detecting protein biomarkers in animal wound fluid.

1.3. Experiment procedure to detect target biomarkers

The experiment is carried out in six steps, with each step lasting 10 minutes (except the antibody step), during which real-time impedance monitoring is performed.

Real-time monitoring of impedance changes in the wireless system is crucial for capturing biological binding events as they occur. The objective is to distinguish the signal from the antibody and the negative control to confirm any differences. After functionalizing the sensor with antibodies and verifying the signal disparity between the negative control and the antibody, the experiment progresses to detecting IL-6. The IL-6 signal is compared to the negative control, and in a subsequent set of experiments, varying concentrations of IL-6 are introduced to the sensor to evaluate differences in the signals generated by the two concentrations of the target protein. The reagents are introduced into the fluidic PDMS cell as previously described. The target biomarker, recombinant mouse IL-6 (406-ML), and the mouse IL-6 antibody (MAB406) were procured commercially (R&D Systems), and all antibody (20 µM) and target biomarker solutions (500 nM and 50 nM) were prepared in PBS. PBS is used in the experiment to provide a stable ionic environment, ensuring consistent impedance measurements and mimicking physiological conditions due to its balanced pH and ion composition similar to bodily fluids, which is crucial for accurate biomarker detection. Fig. S3 illustrates the output voltage of the lock-in amplifier connected to the transmitter side during various steps of the experiment. The experiment is initiated by introducing 10 µl of phosphate-buffered saline (PBS) into the sensor. This stage of the experiment demonstrates a significant increase in output voltage from the baseline when the sensor is unfilled (See Fig. S3 (a)).

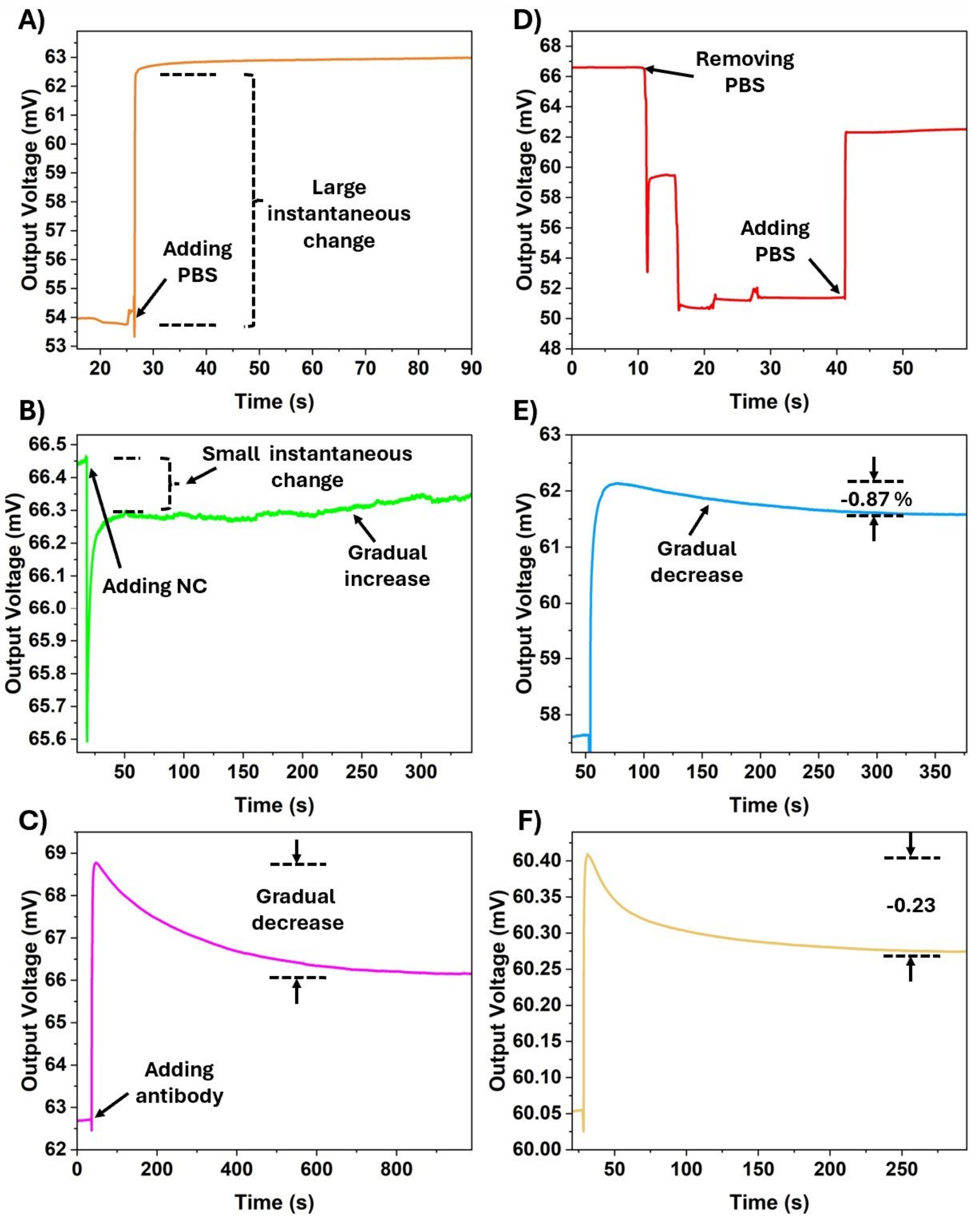

Fig. S3: Experimental procedure using the wireless setup with RFID coils to detect two different concentrations of IL-6: 500 nM and 50 nM (unitless percentage change). These steps include: (A) adding 10 µl PBS to the dry sensor, (B) adding 2.5 µl negative control blank PBS sample, (C) adding 2.5 µl of iL-6 antibody solution to the sensor. (D) Then, the sample is removed from PDMS well and it is refilled with a 10 µl PBS sample. This is followed by adding another 2.5 µl blank PBS sample to the sensor as the negative control (Fig. S4.). Finally, 2.5 µl of iL-6 is added at 500 nM (E) and 50 nM (F) in two different experiments.

The equivalent impedance observed by the lock-in amplifier reflects changes in the overall impedance. During this step, the absolute impedance within the sensor undergoes a dramatic shift, leading to a noticeable change in the net impedance, which is influenced by the sensor's impedance. Due to the high conductivity of the PBS buffer, the sensor's impedance undergoes a significant change. This aligns with expectations, as the introduction of the buffer establishes a conductive path between the two electrodes, unlike when the sensor is empty. The introduction of PBS into the sensor causes a significant shift in the current in the receiver circuit and, consequently, the current delivered to the lock-in amplifier in the transmitter circuit, as the input voltage remains constant. This shift leads to an abrupt change in the lock-in amplifier's output voltage, reflecting the baseline adjustment that occurs immediately after PBS addition. A 2.5 μl blank PBS sample, used as a negative control, is introduced into the sensor. In this step, the introduction of PBS on top of the existing PBS causes a noticeably smaller instantaneous change compared to the previous step, where PBS was introduced to the dry sensor. In addition to this change, a gradual upward trend follows the initial instantaneous change, as illustrated in Fig. S3(B). Subsequently, 2.5 μl of IL-6 antibody is introduced into the well. This leads to the binding of antibodies to the surface of the gold electrodes, and unlike the negative control, this step results in a gradual decrease in the output voltage. This occurs after the initial instantaneous shift in the baseline, as shown in Fig. S3(C). As the antibodies bind to the electrode surfaces, they impede the flow of ionic current between the electrodes. As a result, the resistance sees a change, and this gradual voltage change reflects an exponential pattern in the baseline. This is consistent with the similar exponential trend observed during our wired measurements, which builds upon the previously mentioned instantaneous shift. The time-dependent signal in the sensor arises from antigen-antibody binding kinetics, where association and dissociation rates gradually saturate binding sites. Diffusion-limited transport slows antigen movement to the sensor surface, while conformational changes and multilayer interactions during binding further contribute. Additionally, the sensor response stabilizes over time as charge distribution adjusts, resulting in a gradual signal change. A comparison of the gradual voltage change during the negative control step with the gradual voltage change in the antibody step demonstrates notable differences between the two signals. Comparing the gradual impedance changes from antibody-antigen binding to the negative control confirms their specificity to antigen-antibody interactions, excluding random or non-specific effects. The negative control also minimizes false positives by identifying non-specific signals, ensures sensor stability in the absence of binding, and verifies effective surface functionalization by limiting measurable changes to specific binding events. When antibodies bond to the electrode surfaces, the signal reaches a point where the impedance stabilizes, showing minimal or no gradual changes over time, indicating that the electrode surfaces are approaching saturation, as seen in Fig. S3(C). After sensor functionalization with the IL-6 antibody, we conducted the next steps involving IL-6 proteins. Thus, the excess antibodies are removed from the sensor and 10 μl of PBS is introduced into the well (see Fig. S3(D)). In the next step, 2.5 μl of PBS is added to the existing solution in the well as a negative control, enabling comparison of its corresponding signal with that of the target protein. As observed in Fig. S3(B), this step demonstrates a similar gradual increase in the baseline. A detailed comparison of the gradual changes between the negative control step and the target protein is shown in Fig. S4.

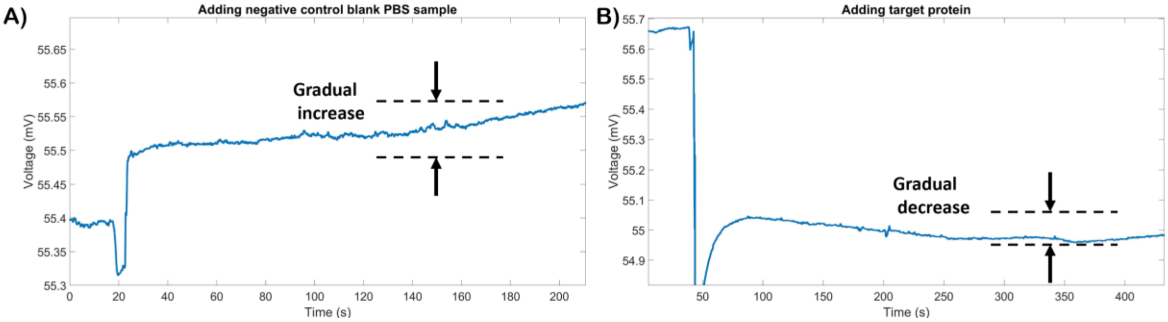

Fig. S4: Comparison of gradual changes in output voltage. (A) The addition of the negative control (blank PBS sample) results in a gradual upward change in output voltage. (B) The addition of IL-6 results in a gradual downward change in output voltage.

Finally, 2.5 µl of IL-6 solution at a concentration of 500 nM is introduced to the sensor. As anticipated, this addition produces a pattern similar to that observed during the antibody step, characterized by a gradual exponential decrease in the output voltage (see Fig. S3(E)). The binding of the target protein to the electrode surface obstructs ionic flow between the electrodes, causing a time-dependent resistance change that can be detected by real-time monitoring of the gradual impedance variation. In a separate experiment, the procedure is repeated up to the addition of the target protein, with the final step involving the introduction of a different concentration of IL-6 (50 nM) to the sensor (see Fig. S3(F)). The observed gradual pattern aligns with that of the target protein step in the previous experiment and the antibody step, exhibiting a time-dependent decrease opposite to the trend observed in the negative control step. However, the gradual change observed in the target protein step of this experiment (0.23%) is significantly smaller than in the previous experiment (0.87%), providing further confirmation that the output voltage in the final step reflects antigen-antibody binding. Additionally, an increase in antigen concentration in the solution leads to a higher number of binding events, resulting in a distinct and measurable change in the gradual impedance of the sensor.

1.4. Animal images

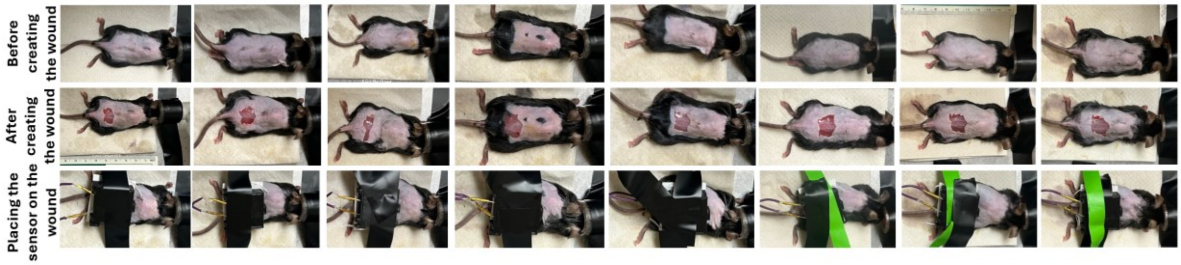

Fig. S5: Eight other animals at different stages: before and after wound creation, and during measurements.

1.5. Output voltage variation over time for low concentrations of IL-6

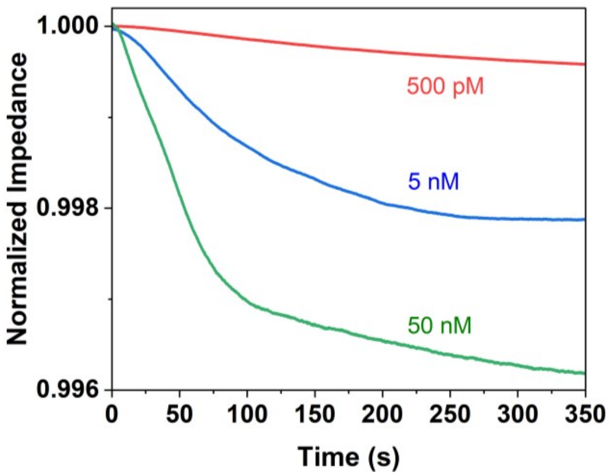

Fig. S6: Output voltage variation over time for IL-6 concentrations of 500 pM, 5 nM, and 50 nM.
